# Characterization of a lytic polysaccharide monooxygenase from *Aspergillus fumigatus* shows functional variation among family AA11 fungal LPMOs

**DOI:** 10.1016/j.jbc.2021.101421

**Published:** 2021-11-17

**Authors:** Fredrik Gjerstad Støpamo, Åsmund Kjendseth Røhr, Sophanit Mekasha, Dejan M. Petrović, Anikó Várnai, Vincent G.H. Eijsink

**Affiliations:** Faculty of Chemistry, Biotechnology and Food Science, Norwegian University of Life Sciences (NMBU), Ås, Norway

**Keywords:** auxiliary activity family 11, AA11, chitin, hydrogen peroxide, lytic polysaccharide monooxygenase, LPMO, substrate specificity, synergy, chitinase, crystal structure, AA, auxiliary activity, AscA, ascorbic acid, H_2_O_2_, hydrogen peroxide, LPMO, lytic polysaccharide monooxygenase, MWCO, molecular weight cutoff, O_2_, oxygen, PASC, phosphoric acid-swollen cellulose, PES, polyether sulfone, ChiC, *Sm*ChiC

## Abstract

The discovery of oxidative cleavage of recalcitrant polysaccharides by lytic polysaccharide monooxygenases (LPMOs) has affected the study and industrial application of enzymatic biomass processing. Despite being widespread in fungi, LPMOs belonging to the auxiliary activity (AA) family AA11 have been understudied. While these LPMOs are considered chitin active, some family members have little or no activity toward chitin, and the only available crystal structure of an AA11 LPMO lacks features found in bacterial chitin-active AA10 LPMOs. Here, we report structural and functional characteristics of a single-domain AA11 LPMO from *Aspergillus fumigatus*, *Af*AA11A. The crystal structure shows a substrate-binding surface with features resembling those of known chitin-active LPMOs. Indeed, despite the absence of a carbohydrate-binding module, *Af*AA11A has considerable affinity for α-chitin and, more so, β-chitin. *Af*AA11A is active toward both these chitin allomorphs and enhances chitin degradation by an endoacting chitinase, in particular for α-chitin. The catalytic activity of *Af*AA11A on chitin increases when supplying reactions with hydrogen peroxide, showing that, like LPMOs from other families, *Af*AA11A has peroxygenase activity. These results show that, in stark contrast to the previously characterized *Af*AA11B from the same organism, *Af*AA11A likely plays a role in fungal chitin turnover. Thus, members of the hitherto rather enigmatic family of AA11 LPMOs show considerable structural and functional differences and may have multiple roles in fungal physiology.

Many microorganisms produce an arsenal of carbohydrate-active enzymes that concertedly operate to degrade carbohydrate biomass. These enzyme systems are fundamental to the global carbon cycle and prevent an environmental buildup of stable and abundant sugar polymers such as cellulose and chitin (1). The discovery of lytic polysaccharide monooxygenases (LPMOs) by Vaaje-Kolstad *et al.* (2) in 2010 changed the idea that depolymerization of cellulose and chitin is accomplished exclusively by hydrolytic enzymes or glycoside hydrolases. It is now well known that many aerobic microorganisms cosecrete LPMOs and glycoside hydrolases during growth on insoluble sugars (3, 4, 5, 6), and the contribution of LPMOs to the efficiency of chitinolytic or cellulolytic enzyme cocktails is well established (2, 7, 8, 9, 10, 11, 12, 13).

LPMOs have gained substantial interest from industries and the scientific community because of their unusual enzymatic properties. Using a single copper ion cofactor, bound in a highly conserved so-called histidine brace (14, 15), LPMOs carry out oxidative cleavage of glycosidic bonds using molecular oxygen (O_2_) (2) or hydrogen peroxide (H_2_O_2_) (16) as a cosubstrate. The redox reaction catalyzed by LPMOs depends on reduction of the bound Cu(II) to form Cu(I) (2, 17). In the case of an O_2_-driven reaction, the reaction requires two externally delivered electrons per catalytic cycle (2). In the case of H_2_O_2_-driven reaction, which tends to be much faster (16, 18, 19, 20), a reduced LPMO can carry out multiple reactions without the need for additional externally delivered electrons (16, 21, 22). Cellulose-active LPMOs oxidize either the C1 or the C4 carbon of the scissile glycosidic bond; some LPMOs exclusively act on C1 or C4, whereas others are less specific and produce a mixture of C1-oxidized and C4-oxidized products (23, 24). This oxidation leads to spontaneous subsequent bond cleavage (25). C6-oxidized products have also been reported (26, 27), but their importance and connection to the chain-cleaving function of LPMOs remain unclear. For chitin-active LPMOs, only C1-oxidizing activity has been reported. The latter activity leads to the generation of new chain ends that are lactones, which spontaneously convert to aldonic acids (2).

Despite a strictly conserved catalytic center, that is, the histidine brace, LPMOs display large sequence diversity and currently populate eight “auxiliary activity” (AA) families in the Carbohydrate-Active enZymes database (http://www.cazy.org/Auxiliary-Activities.html; as of August 2021), a sequence-based database of carbohydrate-active enzymes, namely AA families 9 to 11 and 13 to 17 (28). Fungal LPMOs appear in families 9, 11, 13, 14, and 16. Of these, the cellulose-active fungal AA9 LPMOs are the most abundant and the best studied. Remarkably, while chitin-active LPMOs are commonly found among members of the AA10 family, which lacks fungal enzymes, and while fungi are known for their ability to produce multiple chitinases and degrade chitin (29), little is known about fungal chitin-active LPMOs.

Fungal chitin-active LPMOs have been classified into the AA11 family (30). So far, only three members of this family have been subjected to some degree of functional characterization (30, 31, 32). Available structural information is limited to the incomplete crystal structure of the catalytic domain of a two-domain AA11 from *Aspergillus oryzae* (*Ao*AA11; (30)). Family 11 LPMOs are the most widespread of fungal LPMOs as they occur in Dikarya and most basal fungal lineages (33). The ectomycorrhizal fungus *Choiromyces venosus* contains the highest number of genes (*i.e.*, 14 genes) encoding AA11 LPMOs. AA11s are most prevalent in Ascomycota, where at least one gene encoding an AA11 LPMO has been found in 385 (99.2%) out of the 388 analyzed genomes, and where the average number of genes encoding AA11s is 3.9 ± 1.8 (33). The genome of the ascomycete *Aspergillus fumigatus* (34) contains three genes encoding family AA11 LPMOs, here referred to as *Af*AA11A (UniProt ID: Q4WF00; gene, AFUA_3G03950), a single domain enzyme, *Af*AA11B (UniProt ID: Q4WEH3; gene, AFUA_5G03010), a two-domain enzyme with a C-terminal domain of unknown function, and *Af*AA11C (UniProt ID: Q4WM72; gene, AFUA_6G10930), also a two-domain enzyme with a C-terminal domain of unknown function. Interestingly, a recent in-depth functional study of *Af*AA11B (32), which is very similar to *Ao*AA11 (73% sequence identity for the catalytic domains), showed that this enzyme has low activity on chitin (*i.e.*, much lower than chitin-active bacterial AA10 LPMOs) while being efficient when acting on soluble substrates. This raises questions as to the natural function of AA11 LPMOs.

To gain more insight into the AA11 LPMO family and oxidative chitin conversion by fungi, we have structurally and functionally characterized *Af*AA11A, including studies of this protein's ability to potentiate the action of chitinases. We also assessed whether this AA11 LPMO, like AA9 and AA10 LPMOs, is capable of employing H_2_O_2_ as its oxygen-containing cosubstrate. Next to providing insight into the functionality of family AA11 LPMOs, our study provides the first complete crystal structure of a member of this family.

## Results

### Protein production and deglycosylation

**Figure 1:**
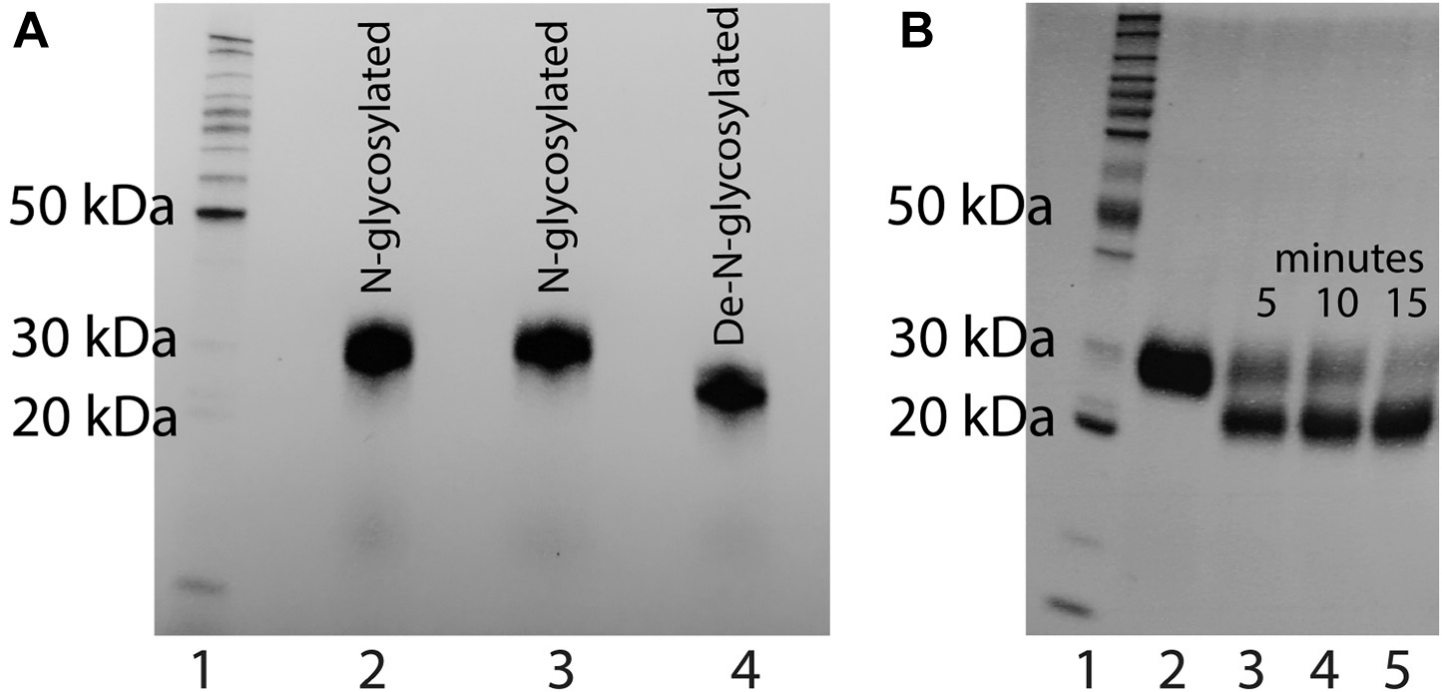
**SDS-PAGE analysis of purified *Af*AA11A with or without deglycosylation.** SDS-PAGE was done with stain-free precast gels and fluorescence imaging. In both panels, lane 1 contains the BenchMark protein ladder. In panel *A*, lanes 2 and 3 show two batches of the N-glycosylated protein, whereas lane 4 shows the protein after overnight deglycosylation with *Ef*Endo18A and subsequent removal of *Ef*Endo18A by chromatography. Panel *B* shows how deglycosylation proceeded in the initial phase of the reaction with *Ef*Endo18A; lane 2, untreated *Af*AA11A; lanes 3 to 5, the protein after treatment with *Ef*Endo18A for 5, 10, and 15 min, respectively. Note that glycosylated *Af*AA11A and *Ef*Endo18A have similar masses and are not separated on this gel.

The gene encoding *Af*AA11A (UniProt ID: Q4WF00) was cloned in *Pichia pastoris*, and recombinant *Af*AA11A was purified to homogeneity from the culture supernatant using hydrophobic interaction chromatography and size-exclusion chromatography (Fig. 1). SDS-PAGE analysis showed that purified *Af*AA11A had a mass close to 30 kDa, indicating that the protein carried glycan moieties. Analysis with the NetNGlyc server (http://www.cbs.dtu.dk/services/NetNGlyc/) showed one possible N-glycosylation site, at Asn62. Indeed, treatment of the purified protein with an endo-β-*N*-acetylglucosaminidase from *Enterococcus faecalis* (*Ef*Endo18A) (35) led to a reduction in the protein mass to a value, approximately 22 kDa, that corresponds well with the theoretical mass (21,750 Da; Fig. 1). Initial comparison of the glycosylated and deglycosylated protein batches indicated similar activities and properties, although minor, borderline significant, differences were observed in, for example, substrate binding and melting point (not shown). Here, we report the functional characterization of the glycosylated protein.

### AfAA11A crystal structure

Crystallization conditions were screened for both N-glycosylated and de-N-glycosylated *Af*AA11A. Only one of the conditions tested resulted in a protein crystal suitable for structure analysis, for the de-N-glycosylated protein. The structure of *Af*AA11A was solved by X-ray crystallography, and a complete structure was obtained, which, however, lacked a bound metal ion in the histidine brace. Crystal data, as well as data collection and refinement statistics, are provided in Table 1. Of note, except for one GlcNAc attached to Asn62, the structure of this N-deglycosylated protein showed no other signs of N-glycosylation or O-glycosylation. Asn62 is not part of the flat LPMO surface containing the catalytic site and is located far away from the catalytic histidines; the Cβ–Cβ distances for Asn62 and His1 and His71 are 21.3 and 22.2 Å, respectively, with side chains pointing into opposite directions.

**Table 1:** Crystal data, data collection, and refinement statistics

Crystal data	

	*Af*AA11A
Space group	P 2_1_2_1_2_1_
Crystal parameters	*a* = 40.99 Å, *b* = 47.28 Å, and *c* = 115.59 Å
	*α* = 90°, *β* = 90°, and *γ* = 90°
Data collection	
X-ray source	MAXIV, BioMAX
Resolution (Å)^a^	29.89–1.50 (1.53–1.50)
Wavelength (Å)	0.946444
Temperature (K)	100
Number of unique reflections	36,807 (1849)
Completeness^a^	99.8 (99.8)
Redundancy^a^	12.3 (8.3)
CC half^a^	0.999 (0.986)
*I*/σ(*I*)^a^	19.2 (3.5)
*R*_merge_^b^	0.061 (0.361)
Refinement statistics	
*R*_cryst_^c^	0.146
*R*_free_^d^	0.197
Wilson *B*-factor (Å^2^)	28.9
Ramachandran plot, in most favored/other allowed regions (%)	98/2
Added waters	182
Protein Data Bank code	7P3U

a Values for outer shell are given in parenthesis.

b $R_{\text{merge}}=\frac{\sum \left|I-\left\langle I\right\rangle \right|}{\sum I}$.

c $R_{\text{cryst}}=\frac{\sum \left(\left|F_{obs}\right|-\left|F_{calc}\right|\right)}{\sum \left|F_{obs}\right|}$.

d *R*_free_ is the *R*_cryst_ value calculated on the 5% reflections excluded for refinement.

**Figure 2:**
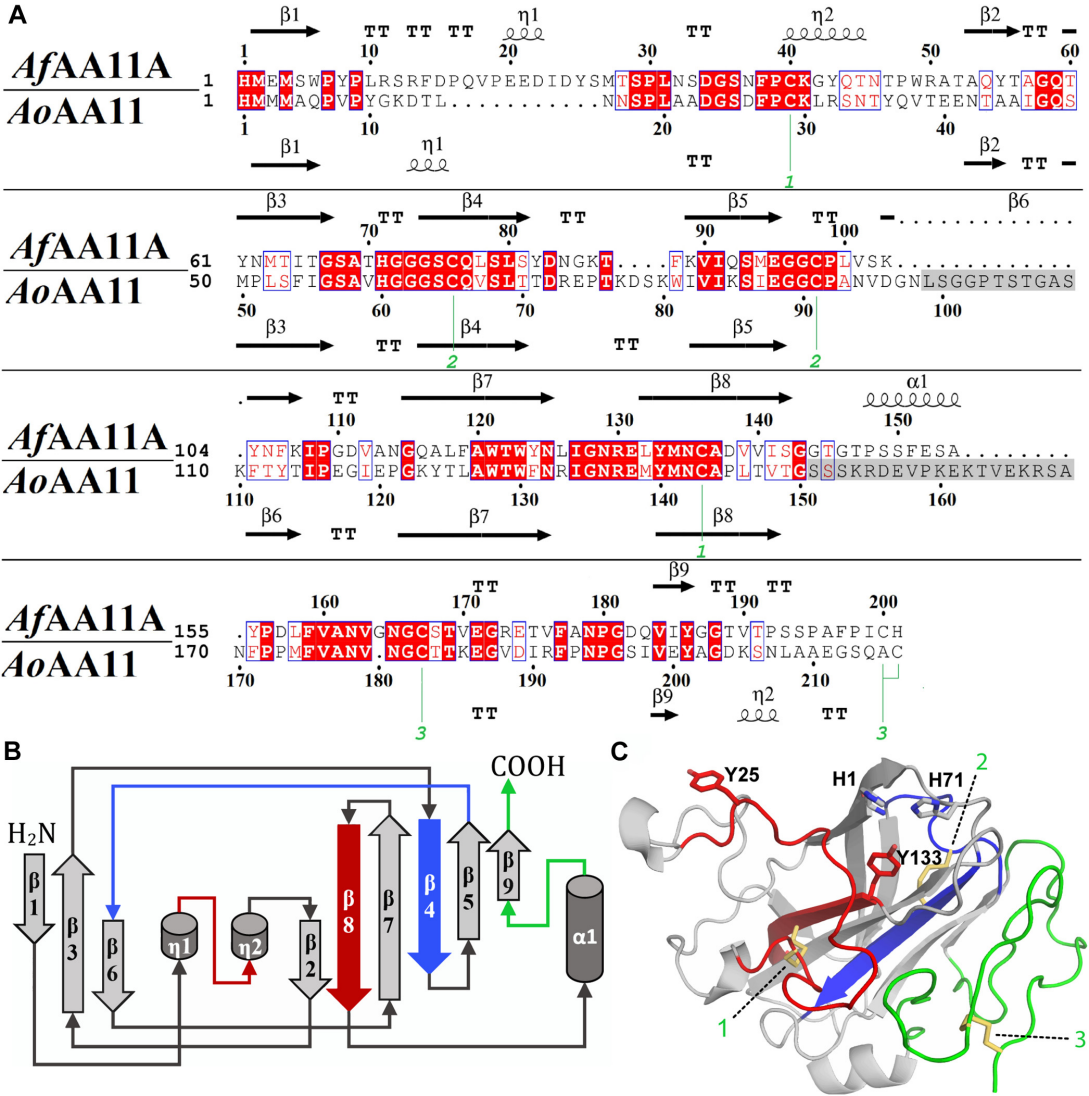
**Pairwise sequence alignment of *Af*AA11A and *Ao*AA11 and structural topology of *Af*AA11A.***A*, structural alignment of *Af*AA11A and *Ao*AA11 (Protein Data Bank ID: 4MAH) generated with the PyMOL alignment tool at default settings. The alignment file was further processed using the ESPript 3 server and Adobe Illustrator. The three pairs of cysteines forming disulfide bridges are labeled with *green numbers*. Residues missing in the crystal structure of *Ao*AA11 are highlighted by *gray shading*. Secondary structure elements are indicated above (*Af*AA11A) and below (*Ao*AA11) the sequence, as follows: *α*, alpha helix; *η*, 3_10_ helix; *β*, beta-strand; T, turn. *B*, topology diagram of *Af*AA11A. *C*, tertiary structure of *Af*AA11A, with the same color coding as in panel *B*; secondary structure elements that are connected by a disulfide bridge (*yellow*; numbering as in panel *A*) appear in the same color (*red*, *blue*, or *green*). Panel *C* also shows the side chains of the two histidines of the copper-binding His brace (H1 and H71), a buried tyrosine beneath the histidine brace (Y133), and a surface-exposed tyrosine (Y25).

**Figure 3:**
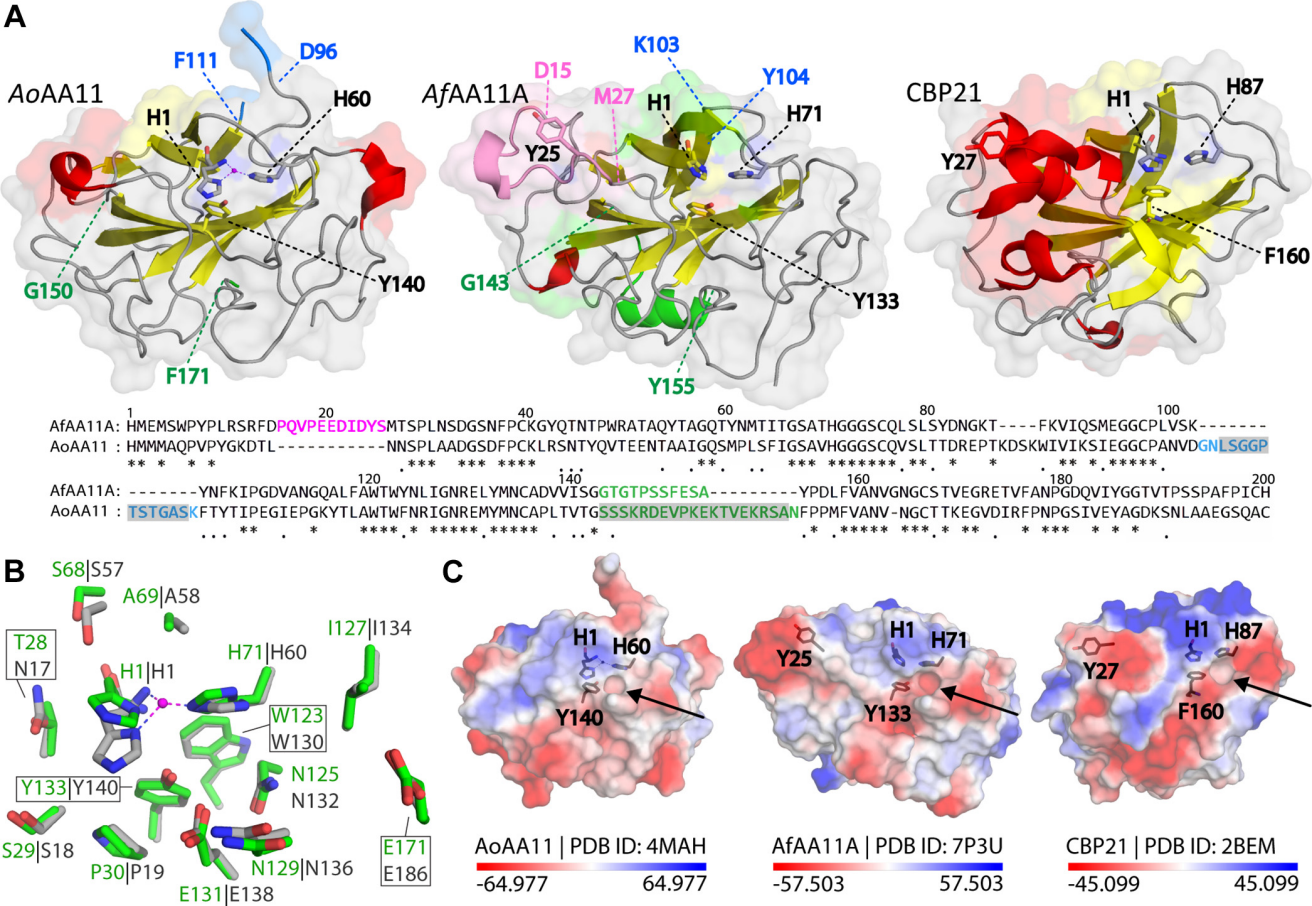
**Comparison of chitin-active LPMOs.***A*, the structures of *Af*AA11A, *Ao*AA11, and CBP21, colored by secondary structure (*helices red*, *strands yellow*) and with additional coloring according to the sequence alignment underneath, showing side chains of residues involved in copper coordination and substrate binding (see text for details). The 11-residue insertion in *Af*AA11A relative to *Ao*AA11 that includes Tyr25 appears in *pink*, and residues adjacent to this insertion are labeled. The 14-residue insertion near residue 100 in *Ao*AA11 appears in *blue*, and residues adjacent to the insertion are labeled in both AA11 structures; note that 11 of these residues (*gray shading* in the sequence) could not be modeled in the *Ao*AA11 structure. Eight of the nine residues forming an insertion near residue 165 in *Ao*AA11 as well as 11 preceding residues could not be modeled in the *Ao*AA11 structure; this 19-residue region is colored *green*, and adjacent residues are labeled. *B*, superposition of *Af*AA11A (*green carbons*) and *Ao*AA11 (*gray carbons*); the picture shows side chains in and near the metal-binding site and a zinc atom bound to *Ao*AA11 (*purple sphere*). *C*, a view on the putative substrate-binding surfaces of the three LPMOs shown in panel *A*, colored by electrostatic surface potential calculated using PyMOL. Note that the *Ao*AA11 structure is incomplete and that the real surface thus may look differently (see main text). The *arrows* point at a small pocket near the catalytic center that seems to be conserved in chitin-active LPMOs, across LPMO families. LPMO, lytic polysaccharide monooxygenase.

The structure of *Af*AA11A (Fig. 2) shows a typical LPMO fold with a core composed of β-strands that are connected by several loops and a few short helices. The core of the protein consists of two β-sheets formed by antiparallel β-strands, which are arranged in an immunoglobulin-like β-sandwich fold. Similar to AA9s, and unlike AA10s, *Af*AA11A has an extended region C terminally of the β8-strand, that is, the most C-terminal strand in the β-sandwich. Pairwise alignments with all structures in the Protein Data Bank using the PDBeFold service (36) at default settings showed that *Af*AA11A is structurally most similar to the catalytic domain of *Ao*AA11 (Protein Data Bank ID: 4MAH), with a Q score of 0.58 and a Cα-RMSD of 1.59 over 186 residues. These two proteins share 48% sequence identity (Fig. 2*A*). *Af*AA11A contains a typical LPMO copper-binding site comprised of the nonmethylated N-terminal histidine and His71 (Figs. 2 and 3). The axial copper coordination positions are occupied by the hydroxyl group of Tyr133 and the methyl side chain of Ala69. This is a common arrangement in LPMOs, including chitin-active AA10s, although the amino acid residues in these axial positions may vary. In particular, bacterial chitin-active AA10 LPMOs have a phenylalanine rather than a tyrosine (Tyr133 in *Af*AA11A) in the proximal axial coordination position. Figure 3*B* shows that the copper-binding regions of *Af*AA11A and *Ao*AA11 are almost identical. The variation in the orientation of His1 is likely because of the absence of a metal ion in *Af*AA11A, since NMR studies have shown that metal binding affects His1 in particular (15, 37).

While the two AA11 enzymes have almost identical catalytic sites, they show considerable differences elsewhere in the structure. Relative to *Ao*AA11, *Af*AA11A has an insertion spanning residues 16 to 26 (Figs. 2*A* and 3*A*), which contains a surface-exposed tyrosine, Tyr25, in a position similar to that of Tyr27 in CBP21 (*Sm*AA10A), a chitin-active AA10 (38) (Fig. 3*A*). Previous studies have shown that this residue is important for chitin binding (38, 39, 40). *Ao*AA11 carries two insertions relative to *Af*AA11A, which both have unknown locations as a major part of both of these is lacking in the *Ao*AA11 crystal structure, as shown in Figure 3*A*. From the location of these insertions, it seems unlikely that they are located in the same area as the Tyr25-containing insertion in *Af*AA11A. Next to most of these two insertions, the *Ao*AA11 structure lacked additional residues (151–161) that are resolved in the *Af*AA11A structure (residues 144–154), showing that these are located far away from the copper-binding site and the substrate-binding surface. Importantly, the insertions in *Ao*AA11 and the additional region that is not visible in the *Ao*AA11 structure contain a tyrosine or other aromatic residues.

Although some uncertainty exists regarding the surface of *Ao*AA11, it is clear that the two AA11 enzymes have very different substrate-binding surfaces, as illustrated in Figure 3*C*, which shows that the substrate-binding surfaces of the two enzymes differ in both shape and electrostatic potential. Interestingly, both AA11 enzymes as well as CBP21 contain a small pocket close to the histidine brace, which has been previously associated with chitin-active LPMOs (41, 42). While it has been proposed that this pocket accommodates an acetyl group (42), recent modeling data suggest that this pocket may give room to O_2_ or H_2_O_2_ in chitin-active LPMOs (43) as in accordance with suggestions by Hemsworth *et al.* (44).

### Substrate specificity

**Figure 4:**
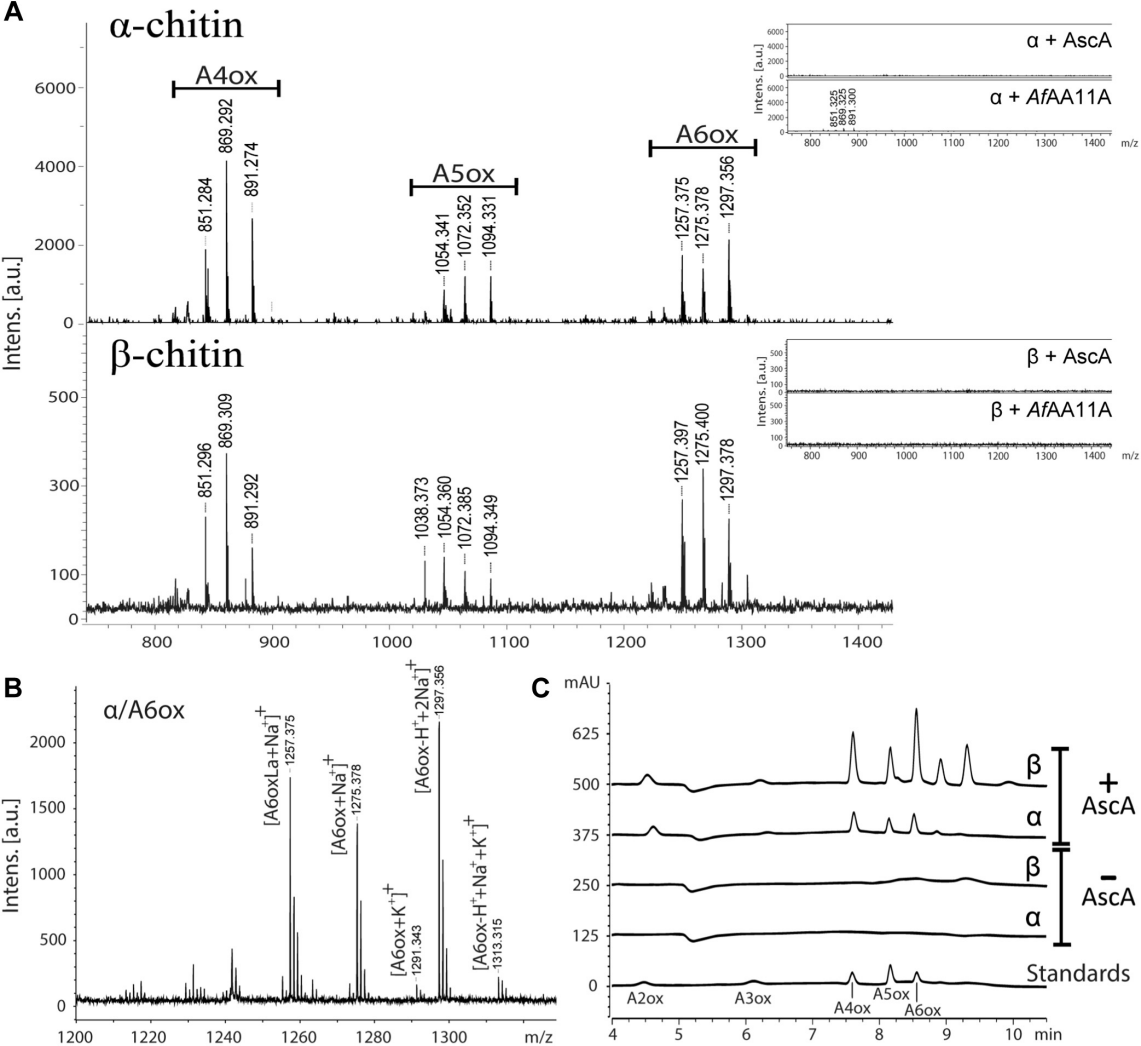
**MALDI-TOF MS and chromatographic analysis of product mixtures generated by *Af*AA11A from chitin.** The reactions contained 1 μM LPMO, 1% (*A* and *B*) or 0.6% (*C*) (w/v) substrate, and 1 mM AscA in 50 mM bis–Tris/HCl buffer, pH 6.5, and were incubated at 30 °C with shaking at 1000 rpm for 24 h. *A*, MALDI-TOF MS spectra showing products generated by *Af*AA11A from α-chitin or β-chitin with AscA; typical clusters of signals that represent oxidized chito-oligosaccharides of different DP are indicated. The *insets* show the negative control reactions, without either LPMO (α + AscA; β + AscA) or AscA (α + *Af*AA11A; β + *Af*AA11A). *B*, detailed view and annotation of the DP6 cluster from the reaction with α-chitin shown in panel *A*. *C*, HILIC analysis of the reaction products; products were identified using a standard containing a mixture of C1-oxidized chito-oligosaccharides with DP 2 to 6. AscA, ascorbic acid; DP, degree of polymerization; HILIC, hydrophilic interaction liquid chromatography; LPMO, lytic polysaccharide monooxygenase.

The substrate specificity of *Af*AA11A was assessed by incubating the LPMO overnight with a variety of substrates in the presence of 1 mM ascorbic acid (AscA). Chromatographic analyses did not reveal any reductant-dependent product formation in reactions with phosphoric acid-swollen cellulose (PASC), Avicel, tamarind xyloglucan, birchwood xylan, beechwood xylan, acetyl glucuronoxylan from aspen, ivory nut mannan, acetylated konjac glucomannan, potato starch, heparin, hyaluronic acid, and chitosan. In contrast, soluble reaction products were observed for the reactions with chitinous substrates (α-chitin from shrimp shell and β-chitin from squid pen), and only if AscA was present (Fig. 4). Both chromatographic and MS analyses of the products generated from α-chitin and β-chitin showed that these were C1-oxidized chito-oligomers with a degree of polymerization 2 to 8 (Fig. 4*C*) (45). The mass spectra showed clusters of signals that are typical for C1-oxidized oligomers, including the diagnostic peak corresponding to the Na^+^ or K^+^ salts of aldonic acids. For example, in the degree of polymerization 6 cluster (Fig. 4*B*), we observed signals corresponding to the Na^+^ adducts of the lactone (*m/z* 1257.375) and aldonic acid (*m/z* 1275.378) forms and of the sodium salt of the aldonic acid (*m/z* 1297.356) as well as the K^+^ adducts of the aldonic acid (*m/z* 1291.343) and of the aldonic acid binding one Na^+^ and one K^+^ ion (*m/z* 1313.315).

### Degradation of chitin in O_2_-driven reactions

**Figure 5:**
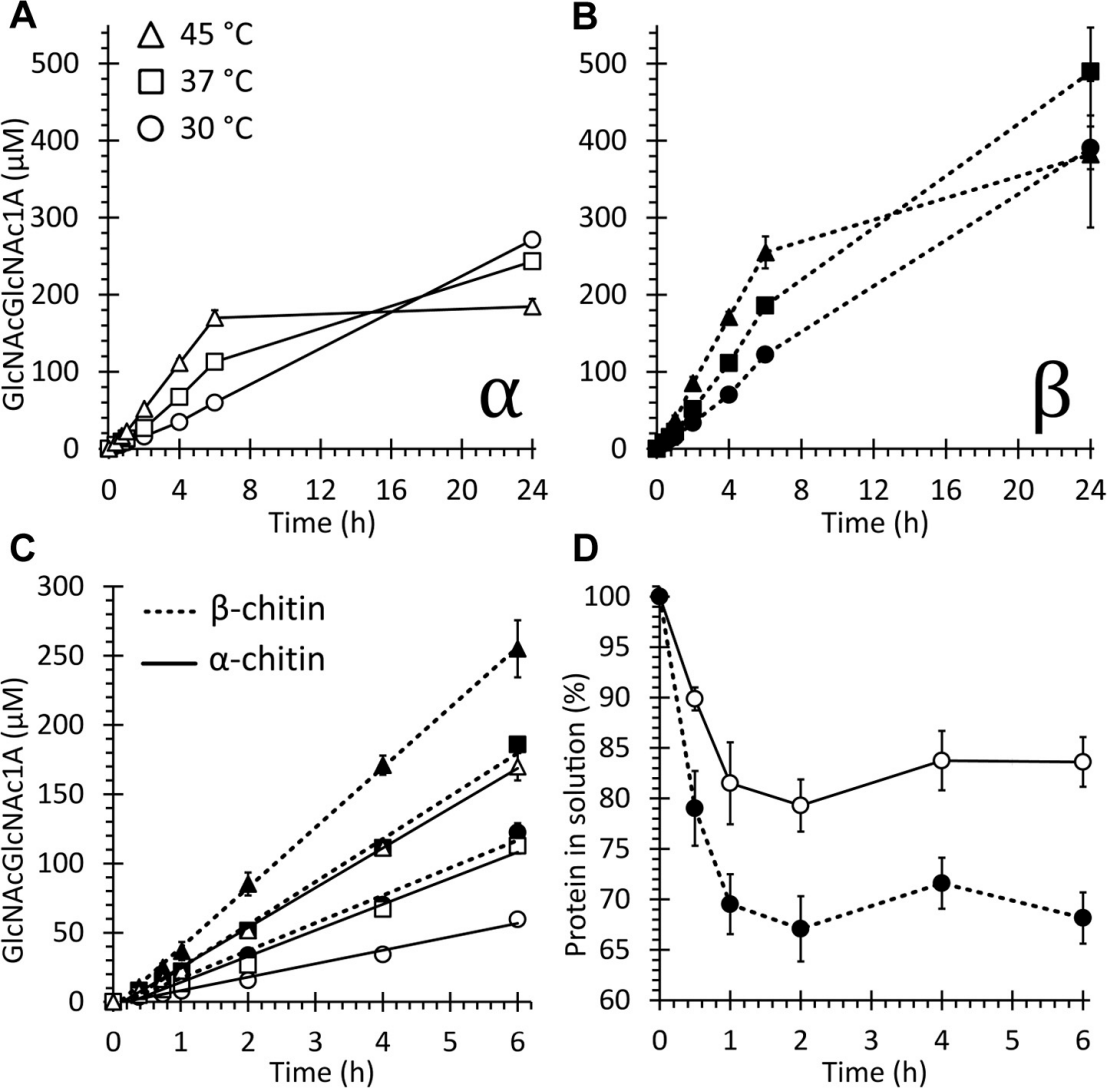
**Activity and binding of *Af*AA11A on α-chitin and β-chitin.** The reactions (*A*–*C*) contained 1 μM LPMO, 1% (w/v) substrate, and 1 mM AscA in 50 mM bis–Tris/HCl buffer, pH 6.5, and were incubated with shaking at 1000 rpm using three different temperatures, as indicated. Before quantification by HPLC, the solubilized reaction products were treated with chitobiase to convert oxidized oligomers to the oxidized dimer (GlcNAcGlcNAc1A). Panels *A* and *B* show progress curves for reactions run at 30, 37, and 45 °C with α-chitin (*A*) or β-chitin (*B*). Panel *C* shows a linear regression of data points for the first 6 h derived from panels *A* and *B*. Binding reactions (*D*) contained 3.0 μM LPMO and 0.2% (w/v) α-chitin (*open circles*) or β-chitin (*filled circles*) in 50 mM bis–Tris/HCl buffer, pH 6.5, and were incubated at 30 °C and 1000 rpm. The graph shows the fraction of the protein remaining free in solution. Error bars indicate standard deviations for three independent reactions. AscA, ascorbic acid; LPMO, lytic polysaccharide monooxygenase.

The ability of *Af*AA11A to degrade α-chitin and β-chitin under conditions that are commonly used when studying LPMOs (aerobic reactions, 1 mM AscA) was initially tested at temperatures between 30 and 45 °C, to find a temperature giving stable reaction kinetics that could be used in subsequent experiments. The product accumulation over time indicates not only faster LPMO rates but also increased nonlinearity, with increasing temperatures (Fig. 5, *A* and *B*). While product formation was linear for the first 6 h at all the three tested temperatures (Fig. 5*C*), only in reactions run at 30 °C, the product formation rate was stable during the full 24 h of the reaction. Hence, all subsequent reactions in this study were run at 30 °C.

Figure 5 also shows that *Af*AA11A is somewhat more active on β-chitin compared with α-chitin, both in terms of the initial rate and the product yield after 24 h. Binding studies showed that *Af*AA11A binds better to β-chitin compared with α-chitin (Fig. 5*D*), which could explain the higher activity toward the former substrate. It is worth noting that the progress curves shown in Figure 5 are similar to those typically obtained with chitin-active bacterial AA10 LPMOs when using similar reaction conditions (*e.g.*, (8)). The low catalytic rates that can be estimated from the curves shown in Figure 5, *A*–*C* (in the range of 0.002–0.01 s^−1^) are commonly observed for LPMOs in AscA-/O_2_-driven reactions (18). Progress curves generated after storing the copper-saturated enzyme for 15 months in 50 mM bis–Tris/HCl, pH 6.5, at 4 °C were similar to those shown in Figure 5, indicating that purified *Af*AA11A had good storage stability.

### Degradation of chitin in H_2_O_2_-driven reactions

**Figure 6:**
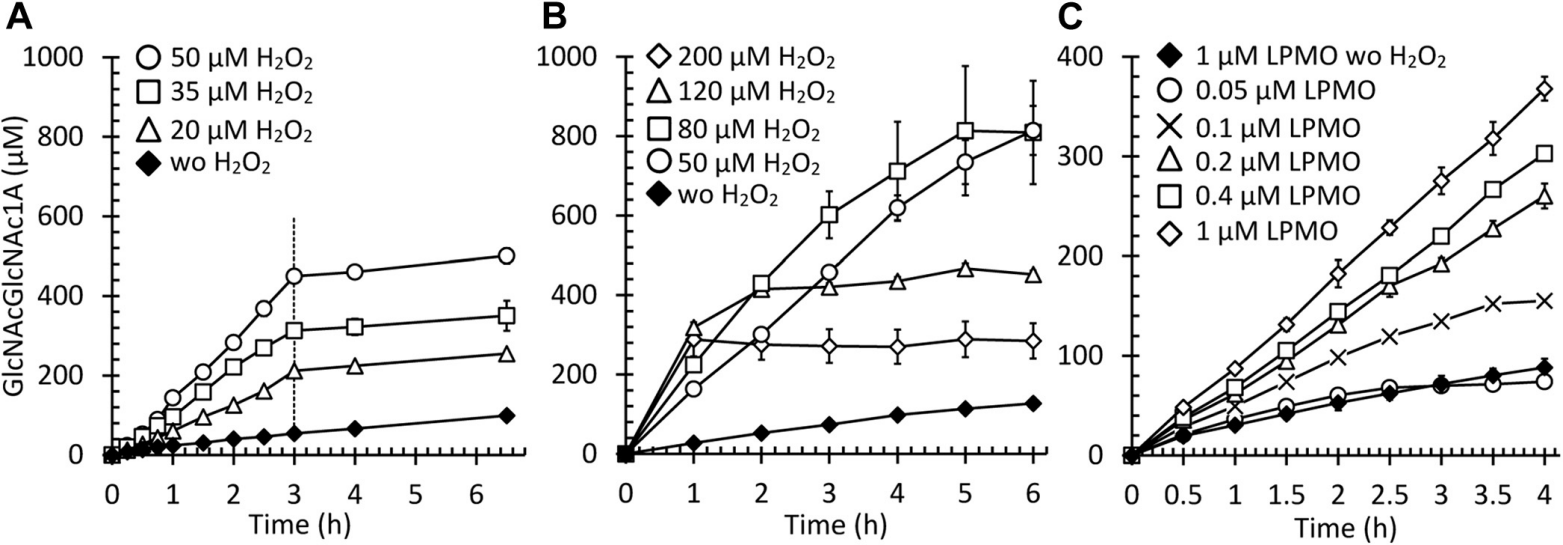
**H**_**2**_**O**_**2**_**-mediated degradation of β-chitin.** Reactions contained 1% (w/v) β-chitin, varying amounts of LPMO, and 1 mM AscA in 50 mM bis–Tris/HCl buffer, pH 6.5, and were incubated at 30 °C with shaking at 1000 rpm. Reactions were supplemented periodically with various amounts of H_2_O_2_ as indicated in the figure and detailed here. At *t* = 0, the reactions were supplied with LPMO, AscA, and then H_2_O_2_. *A*, reactions containing 1 μM LPMO were supplied with varying amounts of H_2_O_2_ (0, 20, 35, and 50 μM) added every 15 min from *t* = 0 up to the 3 h mark (*vertical dotted line*; last addition at 2.75 h). *B*, reactions containing 1 μM LPMO were supplied with varying amounts of H_2_O_2_ (50–200 μM) added every 15 min from *t* = 0 h to *t* = 6 h (Note the fewer sampling points within the first 3 h compared with panel *A*). *C*, reactions containing varying amounts of the LPMO (0.05, 0.1, 0.2, 0.4, and 1 μM) were supplied with 40 μM H_2_O_2_ added every 30 min from *t* = 0 h to *t* = 4 h. Error bars indicate standard deviations derived from three independent reactions. No oxidized products were observed in control reactions lacking AscA or the LPMO (not shown). Reaction products were quantified by HPLC after treatment with chitobiase. AscA, ascorbic acid; H_2_O_2_, hydrogen peroxide; LPMO, lytic polysaccharide monooxygenase.

Recent studies have shown that feeding LPMOs with H_2_O_2_ may lead to fast turnover of chitin or cellulose (16, 20), and one study, describing a novel assay to detect LPMO-generated carboxyl groups in chitin (46), has indicated that this may also be the case for members of the AA11 family. Therefore, we analyzed if and how H_2_O_2_ could be used to boost the catalytic activity of *Af*AA11A. For our experiments, we repeatedly added varying amounts of H_2_O_2_ to reactions containing the substrate, LPMO, and AscA. Thus, we mimicked a situation where H_2_O_2_ is delivered even more gradually, for example, by a H_2_O_2_-generating enzyme (47) or through continuous feeding with a pump, which has shown to be highly effective for cellulose-active LPMOs (16, 48, 49). When H_2_O_2_ was added in lower concentrations (20–50 μM H_2_O_2_; Fig. 6*A*), the accumulation of soluble oxidized products correlated well with the amount of H_2_O_2_ added, indicating that H_2_O_2_ was consumed productively between additions. After the H_2_O_2_ feeding phase (Fig. 6*A*), the LPMO reaction proceeded at the same rate as in the control reaction with only AscA, confirming that H_2_O_2_ had been consumed during the feeding phase and that the LPMO was still active. Increasing the amount of H_2_O_2_ (80–200 μM per addition; Fig. 6*B*) gave higher initial product formation rates combined with increasingly rapid cessation of product formation. The observation that the LPMOs become inactivated when supplied with (too) high amounts of H_2_O_2_ is in accordance with observations made for LPMOs in the AA9 and AA10 families (16, 48).

To get an impression of the maximum rate of H_2_O_2_ consumption by the LPMO, we ran reactions with reduced LPMO concentrations. The results (Fig. 6*C*) show that, at LPMO concentrations below 0.2 μM, the amount of LPMO became limiting for the reaction. On the other hand, dose–response effects were minimal at LPMO concentrations of 0.2 μM and higher, suggesting that in all these reactions, H_2_O_2_ consumption was complete and that H_2_O_2_ was limiting. The apparent initial rates that can be derived from the progress curves for the reactions with 0.05 and 0.1 μM LPMO are in the range of 0.15 to 0.20 s^−1^, which is 1 to 2 orders of magnitude higher than the apparent rates observed in standard reactions with only AscA and no added H_2_O_2_. Of note, the apparent rates of *Af*AA11A are likely underestimated because a significant fraction of the oxidized sites, perhaps up to 50% (50), may remain in the solid residue after saccharification. It is also worth noting that the linear progress curve for the reaction with 0.2 μM *Af*AA11A in Figure 6*C*, where the level of soluble product reaches 250 μM after 4 h, implies that, in this experimental setting, one LPMO molecule can turnover at least some 1250 molecules of H_2_O_2_ without becoming inactivated.

### Synergy with a chitinase

**Figure 7:**
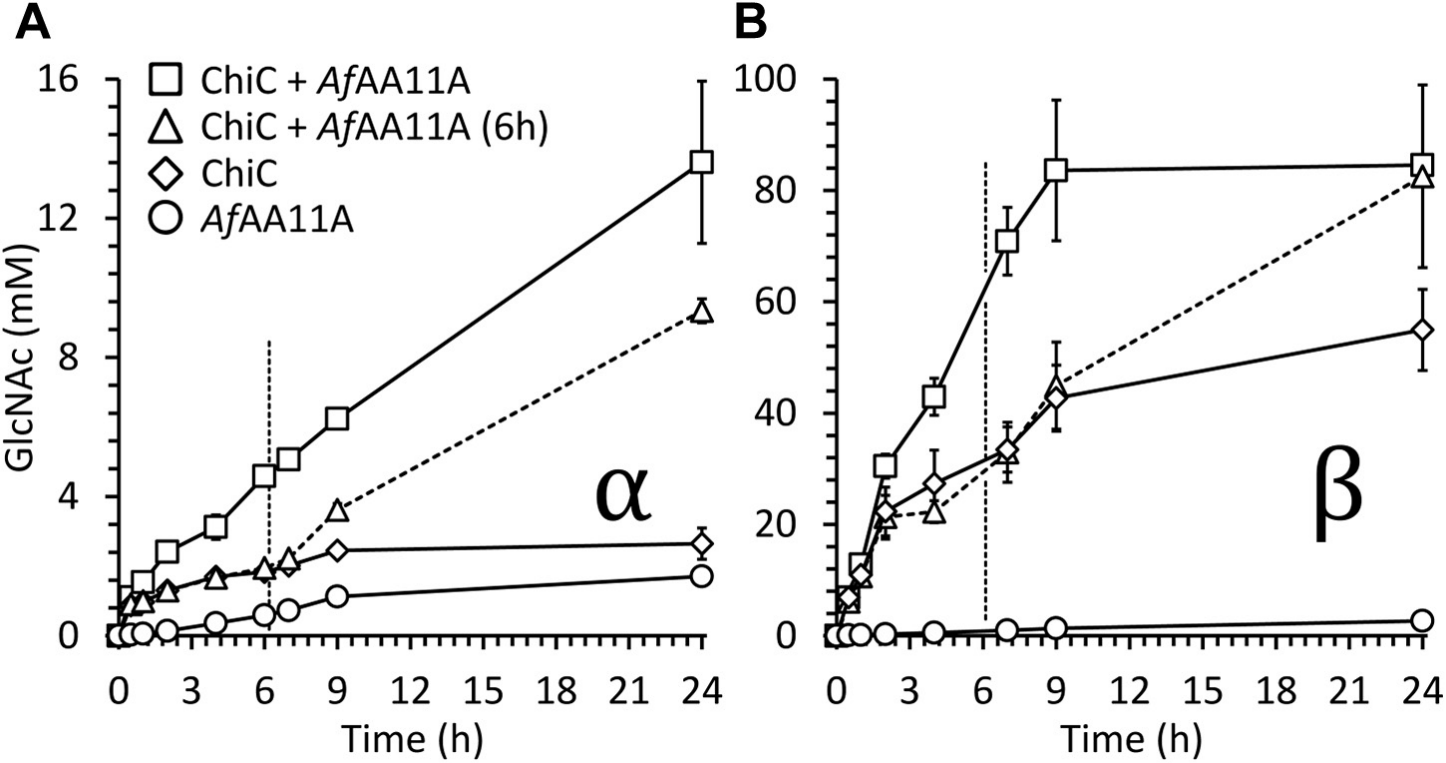
**Synergistic action of *Af*AA11A and a chitinase in chitin degradation.** The figure shows chitin solubilization over time in various reactions with α-chitin (*A*) or β-chitin (*B*). All reaction mixtures contained 1 μM LPMO and/or 1 μM ChiC, and approximately 10 g/l α-chitin or 20 g/l β-chitin in 50 mM bis–Tris/HCl buffer, pH 6.5, and were incubated at 30 °C with shaking at 1000 rpm. For reactions containing the LPMO, 1 mM AscA was included. Before quantification, reaction products were treated with chitobiase to convert all soluble products to a mixture of GlcNAc, which was quantified, and minor amounts of GlcNAcGlcNAc1A. The *vertical dotted lines* indicate the time point for addition of *Af*AA11A in the reactions labeled “ChiC + *Af*AA11A (6 h).” Error bars indicate standard deviations for three independent reactions. AscA, ascorbic acid; ChiC, *Sm*ChiC; LPMO, lytic polysaccharide monooxygenase.

The ability of *Af*AA11A to boost chitinase efficiency was assessed by monitoring the degradation of α-chitin and β-chitin by a combination of an endochitinase, chitinase C from *Sm*ChiC (ChiC), and *Af*AA11A in a 1:1 M ratio (Fig. 7). The results show that *Af*AA11A promotes the activity of *Sm*ChiC on both α-chitin and β-chitin. When acting individually on α-chitin for 24 h (Fig. 7*A*), the proteins were able to solubilize 1.7 and 2.6 mM GlcNAc of the substrate, respectively, while when combined, 12.6 mM GlcNAc was produced. In the reactions with α-chitin and *Sm*ChiC only, product formation stopped after some 9 h, whereas linear product formation was achieved for 24 h in the reaction that also contained the LPMO.

Synergistic effects were also observed in reactions with β-chitin (Fig. 7*B*). Compared with the reactions with α-chitin, these effects were smaller, which may be due to the fact that the chitinase alone was much more efficient on β-chitin (Fig. 7*B*) than on α-chitin (Fig. 7*A*). The initial rate of product formation by *Sm*ChiC was little affected by the addition of *Af*AA11A. On the other hand, in the reaction where *Sm*ChiC was supplemented with the LPMO, the high initial rate (as observed with *Sm*ChiC alone) was maintained for a longer time, and, consequently, higher product levels were reached earlier on in the reaction. Final saccharification yields after 24 h were also affected by the LPMO. While *Sm*ChiC and *Af*AA11A alone were able to produce 54.9 and 2.7 mM GlcNAc, respectively, 84.6 mM GlcNAc was produced in the reaction containing both enzymes.

To highlight the impact of the LPMO in chitin depolymerization, and to verify whether the cessation of product formation in reactions with *Sm*ChiC was caused by enzyme inactivation, denaturation, or depletion of accessible substrate, reactions were carried out in which the LPMO was added after 6 h, instead of at the start of the reaction. This experiment showed that chitin solubilization by *Sm*ChiC was enhanced upon addition of the LPMO (Fig. 7, *A* and *B*) and that, in these reactions, product formation continued for the remaining reaction period. Thus, the gradual cessation of product formation in reactions with only *Sm*ChiC is due to the enzyme not being able to access the remaining substrate and not to enzyme inactivation.

## Discussion

The crystal structure of *Af*AA11A is only the second crystal structure of a member of the widely spread family of fungal AA11 LPMOs and the first one that shows all amino acids. The *Af*AA11A structure lacks a metal ion, which is remarkable considering the reported high metal affinities of these enzymes (14, 15) but not uncommon (*e.g.*, (38)). Nevertheless, the catalytic centers of *Af*AA11A and *Ao*AA11, which has a zinc ion bound, look similar (Fig. 3*B*). Comparison of the structures of *Af*AA11A and *Ao*AA11 beyond the catalytic centers is complicated because the latter structure lacks two regions comprising 30 residues in total. Residues 144 to 154 in the structure of *Af*AA11A provide structural information for residues 151 to 161 lacking in the structure of *Ao*AA11. It is somewhat remarkable that these residues form an α-helix (α1; Fig. 2), since the fact that these residues could not be modeled in the *Ao*AA11 structure would suggest that they are part of a flexible loop-like structure.

Even when taking into account the lacking information for *Ao*AA11, it seems safe to conclude from the comparison of the two structures (Fig. 3) that the two enzymes have different substrate-binding surfaces. Because of the Tyr25-containing insertion that is unique for *Af*AA11A, the substrate-binding surface of this enzyme shows features that are characteristic for chitin-active AA10 LPMOs, such as CBP21, in particular the presence of exposed Tyr25 (Fig. 3). Experimental (38) and modeling (40) studies have shown that a tyrosine in this position is important for binding of crystalline chitin. As alluded to above, it is not possible to envisage a similar structural arrangement in *Ao*AA11 or in previously characterized *Ff*AA11 (31), which both lack this insertion. Interestingly, these structural differences seem to correlate with functional properties. On the one hand, we show here that the catalytic activity of *Af*AA11A and its ability to boost enzymatic chitin conversion are similar to what has been observed for bacterial chitin-active AA10 LPMOs. On the other hand, a recent study has shown that another AA11 LPMO from *A. fumigatus*, *Af*AA11B, which is a close homolog of *Ao*AA11, with similar insertions and deletions relative to *Af*AA11A, has negligible activity toward chitin (32). Of note, *Af*AA11B shows high activity toward soluble chito-oligomers; the biological role of this activity remains enigmatic (32).

Our data show that *Af*AA11A has C1-oxidizing activity leading to production of lactone products that are in equilibrium with their aldonic acid forms. It is worth mentioning that the MALDI-TOF MS analyses suggested the presence of considerable amounts of lactones (M-2 Da) in reactions with both α-chitin and β-chitin, which could represent C4-oxidized products (since C1-oxidized lactones are expected to be hydrated to a larger extent (51)). While this observation, which is similar to observations by Wang *et al.* (31) for *Ff*AA11, could indicate the presence of C4-oxidized reaction products, we did not detect unusual, that is, possibly C4-oxidized products in either hydrophilic interaction liquid chromatography (Fig. 5*C*) or high-performance anion exchange chromatography with pulsed amperometric detection (not shown). Compared with α-chitin, reactions with β-chitin showed both better binding at 30 °C and more product formation at all three tested reaction temperatures (Fig. 5). Other chitin-active LPMOs show similar differences regarding binding to and activity on these two chitin allomorphs (45, 52).

Since the discovery that LPMOs can use H_2_O_2_ as a cosubstrate in 2017 (16), multiple studies have demonstrated that addition of H_2_O_2_ to LPMO reactions leads to increased catalytic rates (16, 20, 22, 46, 48, 53). Indeed, and in accordance with previous observations for *Ff*AA11 (46) and *Af*AA11B (32), we observed increased catalytic rate upon adding H_2_O_2_ to reactions with *Af*AA11A. The apparent catalytic rates of *Af*AA11A were in the range of 0.003 to 0.012 s^−1^ for the O_2_-driven reaction and in the range of 0.1 to 0.2 s^−1^ for the H_2_O_2_-driven reaction. An earlier study on *Ff*AA11A, using the similar reaction conditions (10 g/l α-chitin, 2 μM LPMO, and 1 mM AscA) reported apparent rates of 0.5 min^−1^ (0.01 s^−1^) under standard aerobic conditions without H_2_O_2_ and a 16-fold increase (to 0.11 s^−1^) when 100 μM H_2_O_2_ was supplied (46). Of note, some claim that the so-called “O_2_-driven” reaction under standard aerobic conditions may be limited by the *in situ* generation of H_2_O_2_, which is needed to fuel the LPMO peroxygenase reaction (see *e.g.*, (16, 54), and see (19) for another opinion). The apparent rates for the peroxygenase reaction are much lower than a *k*_cat_ value reported for CBP21 acting on chitin (6.7 s^−1^; (20)). This may reflect a true difference in enzyme efficiency, but it is important to note that the peroxygenase rate of *Af*AA11A is underestimated because of noncontinuous product monitoring, which may hide possibly much higher rates that are achieved right after each addition of H_2_O_2_. Additional kinetic studies are needed to determine the true maximum rate of *Af*AA11A.

The experiments depicted in Figure 6 show that the *Af*AA11A peroxygenase reaction may be limited by H_2_O_2_ (Fig. 6*A*) or by the LPMO concentration (Fig. 6*C*), depending on how the reaction is set up. The H_2_O_2_-limited reactions in Figure 6*A* show a strong correlation between the amount of added H_2_O_2_ and the amount of soluble oxidized products. In each of the three reactions with added H_2_O_2_, the level of oxidized products at the end of the H_2_O_2_-feeding period (and corrected for product levels generated in the control reaction with reductant only) correspond to some 60 to 65% of the total amount of added H_2_O_2_. It is well known from the literature that a considerable portion, in some cases up to 50%, of the oxidized sites introduced by LPMOs remain on the crystalline substrate (55), which may explain the discrepancy between the amount of added H_2_O_2_ and the amount of soluble oxidized products.

As expected for LPMOs (and other enzymes with active redox centers), excessive H_2_O_2_ concentrations lead to enzyme inactivation of LPMOs (16, 56). Figure 6*B* shows that higher H_2_O_2_ concentrations lead to higher initial enzyme rates and more rapid enzyme inactivation, as has been demonstrated for multiple LPMOs, including *Ff*AA11 (46). Importantly, at lower H_2_O_2_ concentrations, the enzyme seems stable, and we show that one enzyme molecule on average catalyzes at least 1250 reactions (note that this by no means is a maximum or an optimized value). This value compares well with previously published values of ≥300 (for *Af*AA11B acting on soluble substrate, (32)) and ≥1500 for the LPMO in a commercial cellulase cocktail (16).

Although the progress curves of Figure 5 (*i.e.*, activity of the LPMO only) showed higher activity of *Af*AA11A on β-chitin compared with α-chitin, the boosting effect of the LPMO on chitinase activity was more pronounced for α-chitin (Fig. 7). α-chitin is more crystalline and resilient to enzymatic degradation than β-chitin (57), and it is thus conceivable that the addition of an enzyme specialized in acting on crystalline material, that is, the LPMO, has a larger effect in reactions with α-chitin. Indeed, previous studies have shown that the extent by which an LPMO can speed up a chitinase reaction depends on substrate crystallinity (58).

In conclusion, we present the first structure of an AA11 LPMO in which all amino acids are included and show that there is considerable structural variation within the AA11 family that is correlated to functional variation. It is worth noting that the disclosed variation within the AA11 family really is remarkable. First, the structural differences in the substrate-binding surfaces seem larger than the differences seen within other LPMO families. Second, while *Af*AA11A degrades chitin with commonly observed reaction rates and yields, *Af*AA11B does not. We also show that *Af*AA11A can efficiently utilize H_2_O_2_ as a cosubstrate and is not inactivated by acting in the “peroxygenase” mode, as long as H_2_O_2_ levels are not too high. *Af*AA11A enhanced the solubilization of β-chitin and α-chitin by *Sm*ChiC, suggesting that this LPMO may have a role in fungal chitin degradation and may be of use in the enzymatic processing of chitin-rich biomass.

## Experimental procedures

### Reagents and substrates

All common reagents used in this study were supplied by Sigma–Aldrich and Merck Millipore, whereas Bacto Peptone and Bacto Yeast Extract were supplied by BD Biosciences, and acetonitrile was supplied by VWR. Alkaline- and acid- pretreated shrimp shell α-chitin originating from *Pandalus borealis* was purchased from Chitinor AS and ball-milled using a Planetary Ball Mill PM100 (Retsch) equipped with a stainless-steel container and zirconium dioxide beads. Milling was performed at 450 rpm in three 5-min rounds with 2-min intervals, yielding a final particle size of ∼0.2 mm. β-chitin originating from squid pen (batch: 20140101) with an average particle size of 0.8 mm was purchased from France Chitine.

Ivory nut mannan, acetylated konjac glucomannan, beechwood xylan, and tamarind xyloglucan were purchased from Megazyme. Birchwood xylan, Avicel, shrimp shell chitosan with approximate mass of 190 to 375 kDa and ≥75% deacetylation, and hyaluronic acid with approximate mass of 0.6 to 1.1 MDa were purchased from Sigma–Aldrich. Potato starch and heparin from pig were purchased from Merck Millipore. Acetyl glucuronoxylan from aspen was produced in-house as described earlier (59). PASC was produced in-house from Avicel, as previously described (60). Tetra-*N*-acetylchitotetraose, penta-*N*-acetylchitopentaose, and hexa-*N*-acetylchitohexaose were purchased from Megazyme. Chitinase *Sm*Chi18C, chitobiase *Sm*GH20 (GenBank ID, AAB03808.1), and endo-β-*N*-acetylglucosaminidase *Ef*Endo18A were recombinantly produced and purified as described previously (35, 61, 62).

*N*-acetyl-d-glucosamine (GlcNAc) with a purity of >95% used for producing standard curves for quantitative product analysis was obtained from Megazyme. Standards of C1-oxidized chito-oligosaccharides were produced in-house by incubating *N*-acetyl-chito-oligosaccharides (Megazyme; 95% purity) with *Fusarium graminearum* chito-oligosaccharide oxidase (*Fg*ChitO) as previously described (62, 63).

### Protein production and purification

The *Af*AA11A protein was produced through heterologous expression using a *P. pastoris* yeast expression system (PichiaPink Strain 4; Thermo Fisher Scientific). A synthetic gene encoding *Af*AA11A (UniProt ID: Q4WF00; gene ID: AFUA_3G03950) including its native signal peptide was codon-optimized for *P. pastoris* and synthesized by GenScript and inserted behind the GAP promoter and a *P. pastoris*-specific Kozak sequence in the pPink-GAP vector by restriction cloning with *Eco*RI and *Acc*65I (New England BioLabs, Inc). The generated plasmid was linearized with *Afl*II (New England BioLabs, Inc) and transformed into electrocompetent *P. pastoris* cells, after which LPMO-producing transformants were screened, selected, and stored as previously described (64).

For protein production, a single colony from a yeast extract–peptone–dextrose agar plate was used to inoculate 50 ml of buffered glycerol complex medium (Invitrogen) in a 250-ml Erlenmeyer flask with vent cap, which was then incubated overnight at 29 °C and 200 rpm. The overnight culture was transferred to 400 ml of fresh buffered glycerol complex medium, followed by incubation overnight in a 2-l baffled shake flask with vent cap. After 24 h, the culture was supplemented with 1% (v/v) glycerol. After 48 h, the culture supernatant containing the LPMO was separated from the yeast cells by centrifugation at 10,000*g* for 20 min at 4 °C and subsequentially filtered through a 0.45 μm polyether sulfone (PES) membrane (VWR). The presence of the LPMO was confirmed by SDS-PAGE gel analysis using Mini PROTEAN TGX Stain-Free precast gels, and fluorescence was recorded with a Gel Doc EZ Imager using the Image Lab, version 6.0.0 Standard Edition software (Bio-Rad). The culture supernatant was concentrated approximately fivefold with concomitant buffer exchange to 50 mM bis–Tris/HCl buffer, pH 6.5, using a Vivaflow 200 tangential crossflow concentrator equipped with a 10,000 molecular weight cutoff (MWCO) PES membrane (Sartorius Stedim Biotech).

For hydrophobic interaction chromatography, the concentrated culture supernatant was supplemented with ammonium sulphate to a final concentration of 2 M and centrifuged for 15 min at 10,000*g* and 4 °C to remove precipitation. The supernatant was then applied onto a 5 ml HiTrap Phenyl FF (HS) column (GE Healthcare Lifesciences), mounted on an ÄKTA Prime Plus chromatography system and equilibrated in 50 mM bis–Tris/HCl buffer (pH 6.5) containing 2 M ammonium sulphate. Proteins were eluted by applying a 35 ml linear gradient toward 50 mM bis–Tris/HCl buffer, pH 6.5. Fractions containing *Af*AA11A were identified by SDS-PAGE, pooled, and concentrated using a Vivaspin 20 Centrifugal Concentrator 10,000 MWCO PES (Sartorius Stedim Biotech). The recombinant protein was further purified by size-exclusion chromatography using a HiLoad 16/600 Superdex 75 pg column (GE Healthcare) mounted on an ÄKTA purifier chromatography system (GE Healthcare), using 50 mM bis–Tris/HCl, pH 6.5, 150 mM NaCl as eluent, and a flow rate of 1 ml·min^−1^. Fractions containing pure LPMO were identified by SDS-PAGE and pooled, followed by concentration and buffer exchange to 50 mM bis–Tris/HCl buffer, pH 6.5, using a Vivaspin 20 Centrifugal Concentrator 10,000 MWCO PES.

To saturate the LPMO with Cu(II), the purified protein was incubated with 10-fold molar excess of CuSO_4_ at 4 °C for 4 h, then desalted on a Vivaspin 20 Centrifugal Concentrator 3000 MWCO PES by five cycles of concentrating and rediluting the protein solution in 50 mM bis–Tris/HCl buffer, pH 6.5 (ca. 10,000-fold dilution). The final protein solution was sterilized by filtration through a 0.22 μm Millex-GV syringe filter (Merck Millipore) and stored at 4 °C.

Protein concentrations of solutions containing pure *Af*AA11A were determined based on absorbance at 280 nm with an Eppendorf Biophotometer D30 (Eppendorf), using the theoretical extinction coefficient of 38,765 M^−1^ cm^−1^, calculated with the ExPASy ProtParam tool (65). The mature protein consists of 201 amino acid residues with a calculated molecular mass of 21,750 Da and a theoretical pI of 4.76 (65).

### Protein crystallization

*N*-linked glycosylations were removed from a batch of purified and copper-saturated *Af*AA11A by treating the LPMO (140 μM) with an in-house–produced recombinant endo-β-*N*-acetylglucosaminidase (*Ef*Endo18A, 3.4 μM; (35)) in 50 mM sodium acetate buffer (pH 6.0). The reaction (with 5 ml total volume) was incubated first at 30 °C for 1 h and then at 4 °C overnight. Deglycosylation was verified by SDS-PAGE, and the *Ef*Endo18A protein was removed by hydrophobic interaction chromatography, as described previously. Fractions containing the LPMO were pooled and concentrated, and the buffer was exchanged to 50 mM bis–Tris/HCl buffer (pH 6.5) using a Vivaspin 20 Centrifugal Concentrator 10,000 MWCO PES. The resulting protein was saturated with Cu(II) and subsequentially desalted, filter sterilized, and stored according to the protocol described previously. Deglycosylated *Af*AA11A was successfully crystallized using conditions 1 to 15 from the commercial crystal screening kit JCSG-plus MD 1-37 Box 1 (Molecular Dimensions) in a hanging drop setup using a 24-well VDX plate with sealant and 18 mm circle glass cover slides (Hampton Research). The mother liquor was made in-house and consisted of 0.1 M bicine (pH 9.0) and 20% (w/v) PEG 6000. The best crystals were produced in droplets obtained by mixing 0.5 μl of 20 mg·ml^−1^ de-*N*-glycosylated *Af*AA11A with 1 μl mother liquor and having 200 μl mother liquor in the well. The crystallization setups were stored in the dark at room temperature, and protein crystals were observed after approximately 3.5 months.

### X-ray crystallography

Protein crystals were soaked in a cryosolution containing mother-liquor with 35% glucose (w/v) before they were flash frozen in liquid nitrogen. Diffraction data were collected at the BioMAX beamline at MAX IV. Datasets were processed by XDS (66) and scaled by AIMLES (67). The CCP4i package was used to solve the structure by molecular replacement (Phaser) (68), and the structure was refined using REFMAC (69). Model manipulations were carried out using Coot (70), and molecular graphics was generated using PyMOL Molecular Graphics System, version 2.0 (Schrödinger, LLC).

### Substrate specificity

The activity of *Af*AA11A was screened toward the following substrates: PASC, Avicel, tamarind xyloglucan, birchwood xylan, beechwood xylan, acetyl glucuronoxylan from aspen, ivory nut mannan, acetylated konjac glucomannan, potato starch, heparin, hyaluronic acid, chitosan from shrimp shell, α-chitin from shrimp shell, and β-chitin from squid pen. Reaction mixtures were set up in 100 μl total volume, contained 1 μM LPMO, 0.2 to 0.6% (w/v) substrate, and 1 mM AscA in 50 mM bis–Tris/HCl buffer, pH 6.5, and were incubated overnight at 30 °C and 1000 rpm in an Eppendorf Thermomixer C (Eppendorf). In negative control reactions, AscA or the LPMO was substituted with Milli-Q water. At the end of the reaction, insoluble substrates were removed by filtration using a 0.2 μm PES 96-well filter plate (Millipore) operated in a vacuum manifold, and the soluble fraction was subsequently analyzed as outlined later.

### Chitin degradation experiments

In general, reactions, with a total volume of 300 μl, were set up with 1% (w/v) substrate and 1 μM LPMO and 1 mM AscA in 50 mM bis–Tris/HCl buffer, pH 6.5, and were incubated at 30 °C and 1000 rpm in thermomixers (Eppendorf Thermomixer C; Eppendorf) in standard aerobic conditions for up to 24 h. Samples (30 μl) were taken periodically, and the reaction was terminated by removing the insoluble substrate by filtration using a 0.2 μm PES 96-well filter plate (Millipore) operated in a vacuum manifold. All reactions were performed in triplicates.

When assessing synergy between chitinases and the LPMO, reactions were set up with various combinations of 1 μM LPMO (*Af*AA11A) and 1 μM *Sm*Chi18C, a nonprocessive endochitinase from *S. marcescens* (61). Reactions containing LPMO were supplemented with 1 mM AscA at *t* = 0. When assessing the effect of H_2_O_2_ on β-chitin degradation by *Af*AA11A, AscA (1 mM) was added at *t* = 0, after which 30 μl of an appropriate aqueous H_2_O_2_ solution was added to reach 20, 35, 40, 50, 80, 120, or 200 μM H_2_O_2_ and a total reaction volume of 300 μl. Reactions were sampled regularly and at the same time supplied with a new portion of H_2_O_2_. Sample volumes (30 μl) were equivalent to H_2_O_2_ addition volumes (30 μl) in order to maintain the reaction volume at 300 μl. Dilution in the amounts of products formed because of sampling and addition of reactant were taken into account when calculating product levels.

All samples were incubated with 1 μM chitobiase (*Sm*GH20) at 37 °C in a static incubator to convert the solubilized oligomers to a mixture of nonoxidized monosugars (GlcNAc) and C1-oxidized dimers (*N,N′*-diacetylchitobionic acid, GlcNAcGlcNAc1A), as described previously (62).

### Substrate binding

To assess binding to chitin, 3.0 μM *Af*AA11A was incubated with 0.2% (w/v) α-chitin or β-chitin in 50 mM bis–Tris/HCl buffer, pH 6.5, at 30 °C and 1000 rpm. At different time points, the insoluble substrate was removed by filtration using a 96-well 0.2 μm PES filter plate (Millipore) installed in a vacuum manifold. The amount of LPMO remaining in solution was visualized on Mini PROTEAN TGX Stain-Free SDS-PAGE gels (Bio-Rad) with a Gel Doc EZ Imager (Bio-Rad) based on fluorescence signals and quantified with the Image Lab software, version 6.0.0. Solutions with known concentrations of *Af*AA11A were used as standard.

### Analysis and quantification of reaction products

Product formation in reactions with cellulosic and hemicellulosic substrates was assessed by high-performance anion exchange chromatography with pulsed amperometric detection using an ICS5000 system (Dionex) equipped with CarboPac PA1 analytical (2 × 250 mm) and guard (2 × 50 mm) columns, using a 50 min gradient protocol as previously described (71, 72). Product formation in reactions with chitinous substrates was assessed by hydrophilic interaction liquid chromatography, using an UPLC (Infinity 1290; Agilent Technologies) equipped with an Acquity BEH Amide Column (130 Å, 1.7 μm, 2.1 × 150 mm) combined with a VanGuard Precolumn (Waters Corporation). The samples were adjusted to 74% acetonitrile prior to analysis, and oxidized chito-oligosaccharides were separated using a 12 min gradient as reported earlier (50). For the identification of produced native and oxidized chito-oligosaccharides, standards were produced as previously described (62). To quantify conversion yields in the chitin degradation assays, soluble products (GlcNAc and GlcNAcGlcNAc1A) were analyzed using a Dionex Ultimate 3000 RSLC system (Dionex) equipped with a Rezex RFQ-Fast Acid H+ (8%) column (7.8 × 100 mm; Phenomenex) operated at 85 °C (8). Data were acquired and analyzed using Chromeleon, version 7.2.9 (Thermo Fisher Scientific).

Oxidized products generated from chitin were also analyzed with MALDI-TOF MS using an Ultraflex MALDI-TOF/TOF instrument (Bruker Daltonics) equipped with a 337 nm nitrogen laser. The samples were mixed in a 1:1 ratio with a matrix solution consisting of 9 mg ml^−1^ 2,5-dihydroxybenzoic acid and 30% acetonitrile and subsequently applied on an MTP 384TF ground steel target plate (Bruker Daltonics). Data acquisition and analysis were performed using FlexControl, version 3.4 and FlexAnalysis, version 3.4 (Bruker Daltonics), respectively.

## Data availability

All data are contained within the article. The crystal structure of *Af*AA11A has been deposited in the Protein Data Bank under the accession code 7P3U.

## Conflict of interest

The authors declare that they have no conflicts of interest with the contents of this article.
